# Microscopic and molecular detection of *Anaplasma* and *Theileria* species in goats from Egypt, with the first report of the emerging zoonotic *Anaplasma capra* and *Theileria orientalis*

**DOI:** 10.3389/fvets.2026.1865200

**Published:** 2026-07-14

**Authors:** Eman Sayed Mohammed, Ismail Elshahawy, Samia Fawy, Marwa Mohamed Fawaz, Qwait AlGabbani, Jamal Asseri, Manal F. El-khadragy, Mustafa Shukry, Ehab Kotb Elmahallawy

**Affiliations:** 1 Department of Parasitology, Faculty of Veterinary Medicine, Qena University, Qena, Egypt; 2 Department of Parasitology, Faculty of Veterinary Medicine, Aswan University, Aswan, Egypt; 3 Department of Biology, College of Sciences and Humanities, Prince Sattam Bin Abdulaziz University, Al-Kharj, Saudi Arabia; 4 Department of Biology, College of Science and Humanities, Shaqra University, Dawadmi, Saudi Arabia; 5 Department of Biology, College of Science, Princess Nourah bint Abdulrahman University, Riyadh, Saudi Arabia; 6 Department of Biomedical Sciences, College of Veterinary Medicine, King Faisal University, Al-Ahsa, Saudi Arabia; 7 Department of Zoonoses, Faculty of Veterinary Medicine, Sohag University, Sohag, Egypt; 8 Departamento de Sanidad Animal, Universidad de Córdoba, Córdoba, Spain

**Keywords:** *Anaplasma*, Egypt, goats, molecular detection, phylogenetic analysis, *Theileria*, tick-borne pathogens

## Abstract

**Introduction:**

Goats constitute a fundamental component of the livestock sector in Egypt, contributing to rural livelihoods, food security, and national income. However, their productivity is increasingly affected by tick-borne diseases. Despite their importance, data on tick-borne pathogens affecting goats in Egypt are still lacking. Anaplasmosis and theileriosis are among the most important tick-borne diseases, posing serious threats to animal health and economic stability.

**Methods:**

This study investigated the microscopic and molecular detection of *Anaplasma* and *Theileria* species in goats from Upper Egypt. Initial screening was conducted by microscopic examination of stained blood smears to identify *Anaplasma* and *Theileria* infections. A total of 60 representative samples, comprising 30 samples for each pathogen, formed a subset based on the presence of high levels of intraerythrocytic infection observed in blood smears and were subjected to molecular analysis. PCR targeting the 16S rRNA gene for *Anaplasma* spp. and the 18S rRNA gene for *Theileria* spp. was performed, followed by sequencing of a subset of representative samples showing the most specific and high-quality amplification products for genetic identification. Additionally, the association between pathogen detection frequency and risk factors was assessed.

**Results:**

Interestingly, microscopic examination of blood smears from 210 goats revealed an overall detection rate of *Anaplasma* and *Theileria* species of 76.19% (160/210; 95% confidence Intervals [CI]: 70.43–81.95). Specifically, *Anaplasma* spp. were detected in 45.71% (96/210; 95% CI: 38.98–52.45) of samples, whereas *Theileria* spp. were detected in 30.48% (64/210; 95% CI: 24.25–36.70). Molecular analysis confirmed the presence of *Theileria orientalis* and *Anaplasma capra*. Statistical analysis demonstrated significant associations between detection frequencies and age and sex. Adult goats (>2 years) showed significantly higher detection of *Anaplasma* spp. (*χ*^2^ = 7.26, *p* = 0.00742) and *Theileria* spp. (*χ*^2^ = 9.889, *p* = 0.0016) compared with younger goats. Likewise, female goats exhibited significantly higher detection of *Anaplasma* spp. (*χ*^2^ = 6.55, *p* = 0.01049) and *Theileria* spp. (*χ*^2^ = 6.519, *p* = 0.01067) compared with male goats.

**Conclusion:**

Overall, this study provides the first molecular evidence of these pathogens in goats in Egypt, including detection of the emerging zoonotic pathogen *Anaplasma capra*. These findings highlight a potential risk to animal and public health and underscore the need for strengthened surveillance and targeted control strategies.

## 1 Introduction

Goats are among the most widely distributed adaptable livestock species, thriving under diverse environmental conditions across all continents and they play a vital socioeconomic role by supporting rural economies, enhancing household food availability and nutrition, and contributing significantly to national agricultural income. In 2023, their population in Egypt was estimated at 4.018 million head. However, this numerical strength is not reflected in production outcomes, as goat productivity remains relatively low. Forecasts covering the period from 2024 to 2028 suggest a gradual decline in performance (1). This gap between population size and productivity may be influenced by multiple constraints, with tick-borne hemopathogens, which are considered a significant factor hindering goat performance. In this respect, goats are known to harbor various ticks that act as vectors and reservoirs of tick-borne pathogens of veterinary and public health importance (2). Among these, theileriosis and anaplasmosis are the most economically important tick-borne pathogens, with a marked impact on goat health and productivity. Theileriosis is a hemoprotozoan infection caused by several species of the genus *Theileria*. At least six species have been reported in small ruminants, with *Theileria lestoquardi* and *Theileria ovis* being among the most commonly identified (3). Anaplasmosis is a rickettsial disease caused by members of the genus *Anaplasma*. To date, seven recognized species have been described, including *Anaplasma ovis*, *Anaplasma marginale*, *Anaplasma centrale*, *Anaplasma platys*, *Anaplasma bovis*, *Anaplasma phagocytophilum*, *Anaplasma odocoilei* and *Anaplasma capra* (4, 5).

Clinically, theileriosis and anaplasmosis in goats can substantially impair goat health and productivity, particularly in endemic regions. *Theileria lestoquardi* infections are commonly associated with fever, enlargement of superficial lymph nodes, progressive anemia, and in severe cases, marked mortality among affected animals. In contrast, anaplasmosis, most frequently caused by *Anaplasma ovis*, is mainly characterized by hemolytic anemia, reduced activity, weight loss, decreased milk yield, and occasional reproductive problems such as abortion. Both diseases may further contribute to immunosuppression, increasing susceptibility to secondary infections and worsening overall clinical performance (6, 7). Notably, anaplasmosis may occur in a subclinical form with minimal or no visible signs, although ongoing hemolysis and systemic effects can still compromise animal health, while clinical cases are characterized by fever, anemia, jaundice, reduced productivity, and other nonspecific signs (8, 9). On top of that, as mentioned earlier, *Anaplasma capra* is an emerging tick-borne zoonotic pathogen that was first detected in blood samples of apparently healthy goats (*Capra aegagrus hircus*) as well as in febrile human cases with a history of tick exposure in China (10–12). It has since been associated with several tick species acting as potential vectors, including *Ixodes persulcatus, Haemaphysalis longicornis, Haemaphysalis qinghaiensis, Dermacentor abaensis, Dermacentor nuttalli, and Rhipicephalus microplus* (10, 13, 14).

In relation to their transmission cycle, ticks of the family *Ixodidae*, commonly known as hard ticks, are obligate hematophagous ectoparasites and are recognized as important biological vectors of a wide range of blood-borne pathogens affecting both animals and humans (15, 16). Within this group, *Hyalomma marginatum* has a broad geographical distribution across Africa, Asia, and parts of Europe, and is well established as a competent vector of several blood-borne pathogens, including species of *Anaplasma* and *Theileria* (16, 17).

Detection of tick-borne pathogens in different animal species in developing countries has mainly relied on microscopic examination for identification of causative agents based on morphology, along with reporting frequency of detection and prevalence. In recent years, however, improving surveillance of tick-borne diseases has increasingly required the adoption of more rapid and accessible diagnostic strategies that can complement conventional laboratory-based methods, particularly in endemic and resource-limited settings (18, 19). In this context, molecular diagnostic techniques represent valuable tools for enhancing pathogen detection and strengthening epidemiological understanding of tick-borne diseases. Among others, Recombinase polymerase amplification combined with lateral flow immunoassay (RPA-LFIA) is widely known as a rapid, isothermal method that enables on-site detection of tick-borne pathogens, thereby complementing PCR-based assays by improving the speed and practicality of molecular diagnostics in field and low-resource settings (20, 21). It should be noted that published data on tick-borne pathogens in goats in Egypt remains limited (22, 23), with virtually no molecular data available from Upper Egypt. This marked gap in knowledge underscores the need for focused investigations to clarify their occurrence and epidemiological significance. Accordingly, the present study aimed to investigate *Anaplasma* and *Theileria* species in goats from Aswan Governorate, Upper Egypt, using microscopic screening followed by molecular confirmation and species-level identification, while risk factors, including age and sex, were evaluated.

## 2 Materials and methods

### 2.1 Ethics approval

The Ethics Committee, Faculty of Veterinary Medicine, Qena University (Egypt), approved the current study for using animals in research [under permit code No.: VM/SVU/23 (2)-27]. The procedures and handling of the animals were carried out in accordance with internationally recognized ethical standards for the care and use of animals in research.

### 2.2 Study area and animal sampling

The current study was conducted in Aswan Province of southern Egypt (coordinates: 24°05′20″N 32°53′59″E) between January 2025 and July 2025. A total of 210 goats of varying ages and sexes were randomly sampled from local household breeders and smallholder livestock keepers, and approximately 3 mL of jugular blood was aseptically collected from each animal into anticoagulated tubes using sterile syringes and needles. The examined goats exclusively exhibited severe emaciation and pale mucous membranes as the predominant clinical findings. Samples were transported to the Parasitology Laboratory, Faculty of Veterinary Medicine, Qena University for further analysis.

Regarding the sample size (n), it was calculated using a conventional prevalence-based epidemiological approach by the following standard epidemiological formula (24):


$$n=\frac{Z^{2}\times P(1-P)}{d^{2}}$$

In this equation, Z was set at 1.96 corresponding to a 95% confidence level, P represented the expected prevalence, and d denoted the desired precision, which is commonly fixed at 0.05. Given the limited baseline information was available for the investigated region, the calculation relied on previously documented infection rates reported in Egyptian goats, including prevalence values of 6.25% and 18.80% for *Anaplasma* spp. and 17.19% for *Theileria* spp. The combined mean prevalence obtained from these studies was used as the expected prevalence parameter in the sample size equation. Assuming a confidence level of 95% and an accepted margin of error of 5%, the estimated minimum sample size was approximately 186 goats. Accordingly, the recruitment of 210 animals was considered adequate to achieve representative epidemiological data and to support the statistical reliability of the study outcomes.

### 2.3 Laboratory analysis

A drop of blood was used to prepare thin blood smears immediately after collection for detection of *Anaplasma* and *Theileria* species. Smears were air dried, fixed in absolute methanol three times for a few minutes, and stained with 30% Giemsa stain for 10 min. Slides were then gently washed under running tap water, air dried, and examined microscopically following Valkiūnas et al. (25). Examination was performed using an Olympus CX31 microscope at 100X magnification for detection of infection. Pathogens were identified according to the descriptions of Soulsby (26). The level of parasitemia was estimated by counting the number of parasitized erythrocytes among 500 erythrocytes examined across five microscopic fields under oil immersion. Parasitemia (%) was calculated using the following formula (27):


$$\text{Parasitemia}=\frac{\text{The amount of blood parasites}}{500}\times 100$$

### 2.4 Molecular and phenotypic investigation

#### 2.4.1 DNA extraction

At this stage, genomic DNA was extracted from a representative subset of 30 blood samples per pathogen that exhibited a high level of intraerythrocytic infection, as determined by microscopic evaluation of stained blood smears. Extraction was done using the GeneJET Genomic DNA Purification Mini Kit (Thermo Scientific), following the manufacturer’s instructions. A 1 kb DNA molecular weight marker (PeqGold 1Kb, Peqlab, GMH) was used to estimate the size of the amplified products. The extracted DNA was stored at −20 °C until further PCR analysis.

#### 2.4.2 PCR amplification

In this phase of the study, PCR was performed on 30 selected samples for detection of *Anaplasma* and *Theileria* species. For *Anaplasma* spp., primers (5′ACACGGTCCAGACTCCTACG 3′ and 5′AGGTACCGTCATTATCTTCCCTACT 3′) were used to amplify a 1299 bp fragment of the 16S rRNA gene (28). For *Theileria* spp., primers (5′ ATTCCCGCATCCTATTTAGCAG 3′ and 5′ CGACTCCTTCAGCACCTT 3′) were used to amplify an approximately 1369 bp fragment of the 18S rRNA gene (28). Briefly, in a 25 μL reaction volume enclosing 12.5 μL of DreamTaq Green PCR Master Mix (2X) (K1081, Thermo Fisher, USA), 1 μL of each primer of 20 pmol, 5.5 μL of water, and 5 μL of DNA. The PCR amplification was conducted in an Applied Biosystem 2720 thermal cycler program as follows: Initial denaturation step of 95 °C for 5 min followed by 36 cycles of denaturation (95 °C for 30 s), annealing (56 °C for 45 s), extension (72 °C for 50 s) and a final extension at 72 °C for 10 min.

#### 2.4.3 Agarose gel electrophoresis and visualization of PCR products

PCR products were electrophoresed on 1.5% agarose gel (Agarose, universal, peq GOLD, peq lab. Germany) in 1x TBE buffer. Approximately 20 μL of the PCR products were loaded onto the gel. Gene marker 50 bp DNA ladder (peq GOLD 2 kb DNA-Ladder, Peq lab., VWR) was used to identify the amplicon size. Gels were stained with a DNA-binding fluorescent dye (0.5 μg/mL) and visualized under ultraviolet light using a gel documentation system (Geldoc-it, UVP, England).

#### 2.4.4 Sequencing and phylogenetic analysis

At this stage, PCR-positive samples were obtained for each pathogen, and 8 representative samples per pathogen were selected for sequencing based on band sharpness, intensity, and expected amplicon size observed by agarose gel electrophoresis. Gel images were documented and analyzed using TotalLab (version 1.0.1)^1^ for band visualization and assessment. Selected PCR products were then purified using a QIAquick PCR Purification Kit (Qiagen, Valencia) and Centri-Sep spin columns prior to sequencing. The DNA sequences obtained by the ABI PRISM^®^ 3,100 Genetic Analyzer (Micron-Corp. Korea) were exposed to BLASTn analysis^2^ performed for sequence identity (29). The phylogenetic tree was constructed using the MegAlign module of Lasergene DNA Star version 1.83 software, based on 16S r RNA gene sequences of *Anplasma capra* and 18S rRNA gene for *Theileria orientalis* (30). The analysis was conducted in MEGA11 using the accession number (OQ236625 for *Anaplasma capra*; OQ236626 for *Theileria orientalis*) with the maximum likelihood method and Tamura-Nei model (31). Bootstrap confidence values were calculated with 500 repetitions. The sequences with host and locality were downloaded from GenBank that was utilized in the tree construction was depicted in Tables 1, 2.

**Table 1:** *Anaplasma* spp. ≥99.83% identity and query cover 100 obtained from GenBank and used in the construction of *Anaplasma capra* phylogenetic tree described in the present study.

Accession number	Species	Host origin	Location/country
OQ236625*	*Anaplasma capra*	Goat	Egypt (the current study)
LC432125	*Anaplasma capra*	Korean water deer	South Korea
LC432123	*Anaplasma capra*	Korean water deer	South Korea
LC432124	*Anaplasma capra*	Korean water deer	South Korea
AB211164	*Anaplasma centrale*	Wild deer and ticks	Japan
AB454075	*Anaplasma* sp.	Deer	Japan
LC432114	*Anaplasma capra*	Water deer	South Korea
MT798599	*Anaplasma capra*	Goat	South Korea
MT799937	*Anaplasma capra*	Goat	China
OM944035	*Uncultured Anaplasma* sp.	Goat	China
OQ148576	*Anaplasma capra*	Goat	China
OR143731	*Anaplasma capra*	Goat	China
MG869594	*Anaplasma capra*	Ticks	China
OQ148549	*Anaplasma capra*	Goat	China
ON399180	*Leucocytozoon sabrazesi*	Pigeon	Egypt

≥ meaning the percentage of identity is greater than or equal to the stated value. The symbol “*” indicates a gene accession number of the present work.

**Table 2:** *Theileria* spp. ≥ 98% identity and Query cover > 98.52% obtained from GenBank sequence and used in the construction of *Theileria orientalis* phylogenetic tree identified in the current study.

Accession number	Species	Host origin	Location/country
OQ236626*	*Theileria orientalis*	Goat	Egypt (current study)
LC602478	*Theileria orientalis*	Dog	Myanmar
MT889728	*Theileria orientalis*	Cattle	Korea
LC576820	*Theileria orientalis*	Cattle	Myanmar
LC576821	*Theileria orientalis*	Cattle	Myanmar
AB520956	*Theileria orientalis*	Cattle	Australia
U97051	*Theileria* sp.	Cattle	Korea
HM538222	*Theileria orientalis*	Buffalo	China
MF576178	*Theileria orientalis*	Bovine	Bangladesh
OR068053	*Theileria orientalis*	Buffalo	India
MG599099	*Theileria orientalis*	Cattle	Pakistan
KT367870	*Theileria annulata*	Cattle	India
MG599097	*Theileria orientalis*	Cattle	Pakistan
OR068050	*Theileria orientalis*	Bovine	India
OM066205	*Theileria orientalis*	Cattle	Turkey
MH208641	*Theileria orientalis*	Ticks	China
ON399180	*Leucocytozoon sabrazesi*	Pigeon	Egypt

≥ meaning the percentage of identity is greater than or equal to the stated value.

### 2.5 Statistical analysis

Statistical analysis was conducted to evaluate the frequency of detection of *Anaplasma and Theileria* species in goats based on epidemiological data. The chi-square (*χ*^2^) test was performed using IBM SPSS Statistics for Windows, Version 21.0 (IBM Corp., Armonk, NY, USA). A significance level of *p <* 0.05 was considered statistically significant (32).

## 3 Results

### 3.1 Frequency of detection of *Anaplasma and Theileria* species and associated risk factors

Microscopic examination of blood smears from 210 goats revealed an overall detection rate of 76.19% (160/210; 95% CI: 70.43–81.95) for *Anaplasma and Theileria* species. Regarding individual detection rates, *Anaplasma* spp. were identified in 45.71% (96/210; 95% CI: 38.98–52.45) of samples, while *Theileria* spp. were detected in 30.48% (64/210; 95% CI: 24.25–36.70).

Regarding the associated risk factors, a significant association was observed between host-related factors and the frequency of detection of both detected pathogens. Age was significantly associated with infection status for *Anaplasma* spp. and *Theileria* spp. (*χ^2^* = 7.26, *p* = 0.007; and *χ^2^* = 9.89, *p* = 0.0017, respectively), with adult goats showing higher detection frequencies of *Anaplasma* spp. (54.54%) and *Theileria* spp. (40.0%) compared with younger animals (36.0 and 20.0%, respectively). Similarly, sex was significantly associated with detection frequency for both pathogens (*χ*^2^ = 6.55, *p* = 0.0105; and *χ*^2^ = 6.52, *p* = 0.0107, respectively), where females exhibited higher frequencies of *Anaplasma* spp. (53.33%) and *Theileria* spp. (37.50%) than males (35.55 and 21.11%, respectively), as summarized in Table 3.

**Table 3:** Association between risk factors and frequency of detection of *Anaplasma and Theileria* species.

Variable	Category	Examined (*n*)	Negative *n* (%)	Positive *n* (%)	*X^2^*	*p*-value	Odd ratio 95% CI	Significance
Age (*Anaplasma* infection)	< 2 years	100	64(64)	36 (36)	7.26	0.007	2.133 (1.225–3.715)	S
	>2 years	110	50(45.45)	60 (54.54)				
Age (*Theileria* infection)	<2 years	100	80(80)	20 (20.0)	9.89	0.0017	2.071 (1.182–3.631)	HS
	>2 years	110	66(60)	44 (40.0)				
Sex (*Anaplasma* infection)	Male	90	58(64.44)	32 (35.55)	6.55	0.0105	2.667 (1.433–4.962)	S
	Female	120	56(46.66)	64 (53.33)				
Sex (*Theileria* infection)	Male	90	71(78.88)	19 (21.11)	6.52	0.0107	2.242 (1.198–4.196)	S
	Female	120	75(62.5)	45 (37.50)				

X^2^ = Chi^2^, p-value = is considered significant at <0.05. Data are presented as numbers and percentages. Statistical analysis was performed using the Chi-square test. S, significant (*P <* 0.05), HS, highly significant (*p <* 0.01).

### 3.2 Morphological description of microscopically detected *Anaplasma* and *Theileria* spp

Giemsa-stained blood smears revealed characteristic morphological patterns for the detected pathogens. In this concern, *Anaplasma* spp. appeared as small basophilic inclusions located at the periphery of erythrocytes, typically observed as single marginal bodies. In contrast, *Theileria* spp. were identified as intraerythrocytic forms, occurring singly or in clusters and exhibiting variable shapes including round, comma-like, and vacuolated forms (Figure 1). Microscopic examination revealed a higher parasitemia level of Theileriosis (9%) compared to Anaplasmosis (7%).

**Figure 1:**
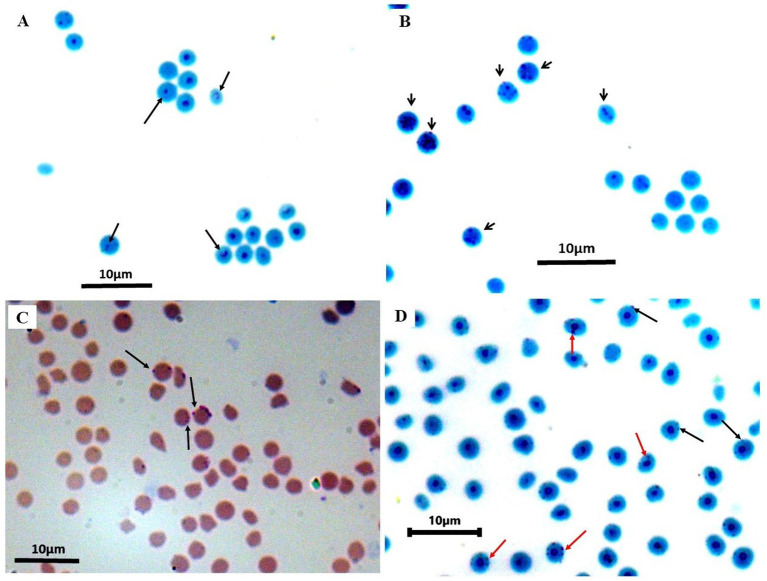
Micrographs of stained thin blood smears of goat stained with Giemsa illustrating intra-erythrocytic inclusions of blood pathogens: **(A)** different forms of *Theileria orientalis* including rounded, vacuole, dot, and comma forms (arrow); **(B)** multiplication of *Theileria orientalis* merozoites inside erythrocytes (head arrow); **(C)** *Anaplasma capra* located peripherally within erythrocytes (arrow); and **(D)** mixed infection with *Theileria orientalis* (red arrow) and *Anaplasma capra* (black arrow).

### 3.3 Genetic characteristics of *Anaplasma and Theileria* species/subtypes

In the present study, molecular analysis of a subset of 30 blood samples per pathogen, selected based on marked intraerythrocytic infection observed microscopically, confirmed the presence of *Anaplasma* and *Theileria* species, thereby validating the microscopic findings. In this regard, amplification of an approximately 1,299 bp fragment of the 16S rRNA gene confirmed the presence of *Anaplasma* spp., while amplification of an approximately 1,369 bp fragment of the 18S rRNA gene indicated the presence of *Theileria* spp. All PCR-positive samples exhibited clear and specific amplification of the expected target amplicons. Subsequent sequencing of 8 representative PCR-positive samples per pathogen showed identical nucleotide sequences within each group across the analyzed gene regions, suggesting low genetic variation among the detected isolates in this study population. The obtained sequences were deposited in the GenBank database under accession numbers OQ236625 (*Anaplasma capra*) and OQ236626 (*Theileria orientalis*). Sequence and BLAST analyses confirmed that the obtained isolate belonged to *Anaplasma capra* (OQ236625). The generated sequence showed 100% nucleotide identity with *A. capra* isolates recovered from Korean water deer in South Korea (LC432123, LC432124, and LC432125), while 99.92% similarity was observed with *Anaplasma* isolates reported from deer and ticks in Japan (AB454075 and AB211164). Phylogenetic analysis further demonstrated that the Egyptian isolate clustered closely with the Korean *A. capra* isolates, supporting their close genetic relationship, as shown in Figure 2. The obtained amplicon sequence of *Theileria orientalis* (OQ236626) showed 99.85% nucleotide identity with *T. orientalis* isolates recovered from cattle in Myanmar (LC576821), while 99.64% similarity was observed with isolates from Indian buffalo (OR068053), dogs in Myanmar (LC602478), cattle in Myanmar (LC576820), and cattle in Australia (AB520956). Furthermore, phylogenetic analysis demonstrated that the Egyptian isolate clustered closely with the Myanmar cattle isolate (LC576821) with strong bootstrap support (95), indicating a close genetic relationship, as shown in Figure 3. Bootstrap was performed using 500 bootstrap replicates, confirming strong evolutionary relationships with global isolates and providing a publicly accessible resource for future molecular and epidemiological investigations.

**Figure 2:**
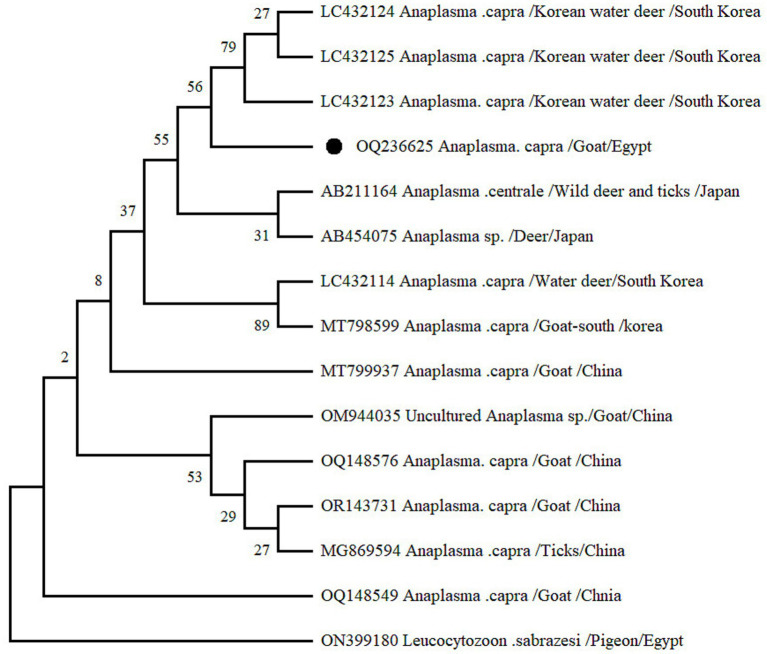
A phylogram of the maximum likelihood analysis of the 16S r RNA gene sequences of *Anplasma* spp. infecting Goat, depicting a strong correlation of the currently identified isolates of *Anplasma capra* marked by black circles (OQ236625). The Maximum Likelihood Tree was implemented in MEGA 11 using *leucocytozoon sabrazesi* as outgroup. This analysis involved 15 nucleotide sequences.

**Figure 3:**
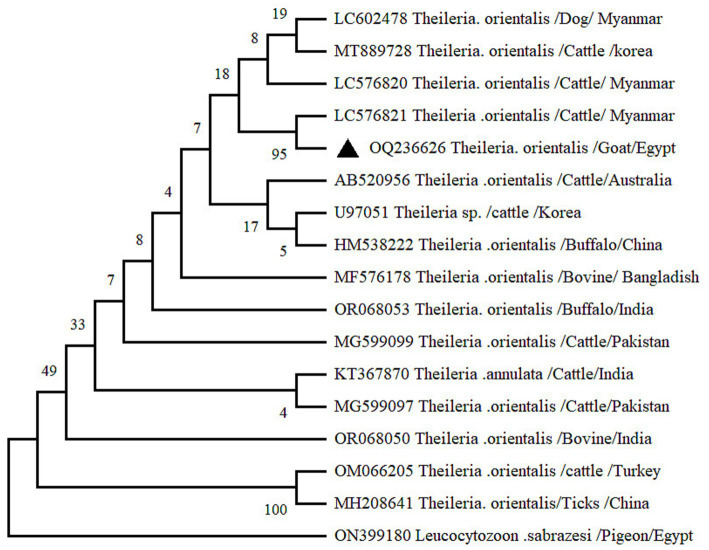
A phylogram of the maximum likelihood analysis of the 18S r RNA gene sequences of *Theileria* spp. infecting Goat, depicting a robust link of the currently identified isolates of *Theileria orientalis* marked by black triangle (OQ236626). The Maximum Likelihood Tree was performed in MEGA 11 using *leucocytozoon sabrazesi* as outgroup. Bootstrap confident values were calculated with 500 repetitions. This analysis involved 17 nucleotide sequences.

## 4 Discussion

Tick-borne pathogens, particularly *Anaplasma* and *Theileria* species, remain among the most important constraints affecting small ruminants and livestock productivity in Egypt (22). In the present study, a considerable burden of infection was detected in goats in Upper Egypt, with molecular and microscopic analyses confirming the concurrent circulation of both pathogens within the examined population. The agreement between diagnostic approaches further strengthens the reliability of these findings and highlights the value of integrated diagnostic strategies in field epidemiological investigations (33). These pathogens are associated with significant health deterioration, reduced production efficiency, and economic losses in animal husbandry systems, consistent with previous reports (34–36). This study provides novel evidence of a considerable burden of tick-borne pathogens in goats in Upper Egypt, revealing the concurrent circulation of *Anaplasma* and *Theileria* species within the population. Integrated microscopic and molecular diagnostics yielded consistent and confirmatory findings, highlighting the strength of combined approaches in field epidemiological investigations.

In the current study, the frequency of detection of anaplasmosis was 45.71% (96/210; 95% CI: 38.98–52.45), indicating a moderate level of circulation of the pathogen within the study population. These results were nearly close to those reported in Indonesia, where the prevalence of anaplasmosis in goats was 47%) (37), and in another study from Indonesia (38), it was 40%, while in the Philippines, the prevalence was 38.64% (39). At the national level, the detection rate observed in the present study exceeds previous reports from Egypt, which documented rates of 18.80 and 6.25% (40, 41). In a broader context, lower prevalence rates of anaplasmosis in goats have been reported in several countries, including Saudi Arabia, where the prevalence was 19.5% (42), Nigeria with 3.05% (43), Thailand with 1.5% (44), Pakistan with 7.87% (45), Bangladesh with 15.75% (46), and China with 30.1% (47). On the other hand, markedly higher infection rates have been reported in Bangladesh (81.82%) and Sudan (60.1%) by Chowdhury et al. (48) and Lee et al. (49), respectively. The variation in prevalence rates observed between the current study and previous reports may be associated with differences in environmental conditions, tick distribution, animal management systems, and diagnostic approaches (22, 40). The comparatively higher prevalence identified in this study may indicate favorable ecological conditions for tick vectors and inadequate implementation of tick-control measures in the investigated area (22, 40). In addition, variations in sample size, geographical origin, and molecular detection methods may further contribute to the differences in prevalence reported worldwide (22, 40). Overall, these findings emphasize the need for ongoing epidemiological monitoring and improved tick-control strategies to limit the spread of tick-borne pathogens in goat populations.

In a similar context, our results recorded an overall theileriosis frequency of 30.48% (64/210; 95% CI: 24.25–36.70), closely aligning with earlier observations from Egypt, where Radwan and Elkelesh (50) reported 28%, and from India, where Arif et al. (51) documented 32.4%. Lower detection rates than those observed in the present study were reported in Indonesia, where Nugraheni et al. (37) recorded 23%, in Thailand with 10.3% (44), in Egypt with 17.19% (41), in Nigeria with 9.5% (52), and in Pakistan with 8.8% (45). In contrast, higher prevalence rates were reported in Thailand at 41.3% (53), Myanmar at 33.3% (54), and lower prevalence were reported in Bangladesh at 8.5% (55), and Uganda at 13.4% (56), indicating notable national and regional variability in the occurrence of the disease. The relatively elevated burden of both anaplasmosis and theileriosis observed in the present investigation may be linked to the environmental characteristics of the Aswan Governorate, where persistently high temperatures, short winter periods, and prolonged summers create favorable ecological conditions for tick survival and multiplication, thereby sustaining continuous transmission of blood-borne pathogens (51). In a broader epidemiological context, although infection frequencies differ widely across geographical regions, the overall occurrence of tick-borne haemopathogens in goats remains substantial in endemic settings. These fluctuations are likely shaped by a combination of ecological factors, host susceptibility, husbandry systems, vector control, and variability in diagnostic methodologies (42, 46). Collectively, the relatively high detection levels observed in this study emphasize the ongoing epidemiological pressure exerted by these pathogens on goat populations in Egypt and underline the importance of sustained molecular monitoring to better understand transmission dynamics and support the design of targeted control interventions.

In relation to the studied risk factors, both anaplasmosis and theileriosis infection showed a clear association with the age structure of the studied goats, as demonstrated in Table 3. The results are consistent with those reported by Arif et al. (51), whereas a similar age-linked distribution was reported. In the present study, adult animals exhibited noticeably higher frequencies of detection compared with younger ones, a trend that is generally interpreted as the outcome of prolonged exposure to tick vectors over time together with the cumulative impact of repeated or subclinical infections. A more comprehensive clinical interpretation of such infections would be obtained by combining direct pathogen detection with evaluation of host physiological responses. In particular, the inclusion of hematological profiles and serum biochemical indicators can help reveal functional and systemic changes associated with infection, thereby providing a more informative picture of disease impact than infection status alone (57). Nevertheless, this pattern is not universal, as contrasting results have been documented by Niaz et al. (45), who found that young goats had a higher prevalence of infection than adult goats. However, according to Shah et al. (58) and Abdelsalam et al. (59) found that age and infection rates do not significantly differ. The higher occurrence of anaplasmosis and theileriosis reported in older goats could be attributed to prolonged and repeated exposure to tick-infested environments over time. As goats advance in age, they spend longer periods grazing outdoors, which increases their exposure to infected ticks and consequently elevates the risk of acquiring tick-borne pathogens. This repeated exposure may also contribute to the establishment of chronic or latent infections in adult animals. Variations reported among different studies may result from several factors, including environmental conditions, geographic differences, husbandry practices, vector density, sample population size, and the sensitivity of diagnostic techniques used (47, 60, 61). In addition, the effect of age on the susceptibility of goats to hemoparasitic diseases may differ depending on the level of endemicity within a specific region (62). Consequently, although age was identified as a significant epidemiological factor in the current investigation, its impact is likely influenced by a complex interaction of ecological and management-related factors (62).

Regarding sex-related patterns, a statistically significant difference was observed, with females showing higher detection frequencies than males for both pathogens. In line with our findings, similar trends in sex distribution and prevalence patterns have been reported in Egypt (7), the Philippines (39), and Nigeria (63). Similar observations were also reported by Niaz et al. (45) and Razzaq et al. (64), with a higher prevalence of anaplasmosis and theileriosis in female than male goats in their studies. In contrast, other previous reports (65, 66) found no significant association between goat sex and infection status, indicating that susceptibility to tick-borne pathogens is unlikely to be sex-dependent and might be driven by other external factors such as husbandry conditions, tick abundance and exposure, environmental risk rather than intrinsic host characteristics. The higher detection rates observed in females compared to males in the present study may be explained by the physiological and management-related influences. From a biological perspective, reproductive stages such as pregnancy and lactation impose considerable physiological strain. These stages involve major metabolic requirements, hormonal variation, and periods of reduced immune efficiency, which can weaken resistance to tick-borne pathogens and increase the chance of sustained infection. From a husbandry standpoint, females are generally maintained in herds for longer durations because of their role in reproduction and milk production, whereas males are often removed at earlier ages (67, 68). This extended retention results in greater cumulative exposure to tick-infested environments and repeated opportunities for pathogen transmission (69). Consequently, females are more likely to acquire and maintain tick-borne infections over time (67, 68).

Based on the available data, this study provides the first detailed molecular investigation of *A. capra* and *T. orientalis* infections in goats in Egypt, offering a valuable baseline for future epidemiological studies on tick-borne pathogens in the region. The *A. capra* sequences obtained here exhibited exceptionally high similarity (99.83–100%) to previously reported sequences in GenBank, showing close genetic relatedness to reference sequences from Korean water deer (LC432125, LC432124, LC432123) (70). Given its zoonotic potential (10), *A. capra* represents an emerging concern with the capacity for cross-species transmission, highlighting the importance of ongoing molecular surveillance to elucidate its epidemiological dynamics. In humans, *A. capra* infection may cause nonspecific clinical manifestations such as fever, headache, and fatigue, particularly among individuals with close and repeated contact with livestock. Moreover, the obtained sequences were compared with all available homologous sequences in the GenBank database. However, the analysis revealed a limited availability of Egyptian reference sequences for *A. capra* and goat-derived *Theileria orientalis* covering the same gene regions. Consequently, the highest sequence similarities were observed with isolates reported from Japan, South Korea, and Myanmar. This pattern likely reflects the current scarcity of molecular epidemiological data from Egypt rather than true phylogeographical relatedness. These findings underscore the relevance of a One Health framework, integrating animal, human, and environmental health sectors, for the control and monitoring of emerging tick-borne diseases. Early molecular diagnosis through PCR-based methods facilitates timely therapeutic intervention and may contribute to reducing inappropriate treatment and the risk of antimicrobial resistance, thereby improving livestock health and productivity. In addition, integrated tick control strategies remain essential for the prevention and effective management of emerging zoonotic tick-borne infections (71, 72).

In our study, sequence analysis provided the first molecular evidence of *T. orientalis* in goats from Egypt, thereby expanding the current understanding of circulating *Theileria* species/subtypes in small ruminants within the country. Notably, the *T. orientalis* sequence retrieved in this study showed 99.85% similarity with a sequence from cattle in Myanmar (LC576821). Although *T. orientalis* is primarily reported in cattle, previous studies have documented its presence in goats and sheep (44, 73). For instance, its presence has been reported in goats from Thailand (44) and in sheep from China (73), with corresponding sequences available under accession numbers OM802550 and KC414098, respectively. This cross-species occurrence may be explained by integrated farming practices, where goats, sheep, and cattle are often raised in proximity, facilitating pathogen transmission through shared vectors. Taken together, the detection of *A. capra* and *T. orientalis* in goats in our study aligns with previous reports from China (47, 74), Thailand (44), and India (75). These findings reinforce the notion that these pathogens are more widely distributed in goat populations than previously assumed, which underscores the importance of sustained monitoring and the implementation of effective control strategies to limit their dissemination and mitigate potential impacts on animal health. Management of tick-borne infections caused by *Theileria* and *Anaplasma* in goats remains complex in endemic settings, largely due to differences in infection timing, host resilience, and clinical expression (76, 77). While commonly used therapies include buparvaquone and oxytetracycline, their effectiveness is often reduced by late detection and the presence of unapparent infections that maintain disease circulation. Continued and unsystematic drug application may further weaken treatment performance, resulting in outcomes that range from unnoticed infection to severe illness and death, reinforcing the importance of early detection and coordinated control measures (76, 77).

A limitation of this work is that only a subset of samples was subjected to molecular confirmation, a decision driven by financial and logistical constraints typical of field investigations in resource-limited settings. This inevitably reduced the scale of molecular screening and may influence the strength of broader epidemiological interpretations. However, such selective testing strategies are commonly adopted in comparable studies where analytical feasibility must be balanced against available resources. A further limitation is the possible under-detection of concurrent infections involving *Anaplasma* and *Theileria*, as the applied diagnostic approach may not fully resolve mixed parasitic presence. While this constraint is acknowledged, the approach remains widely utilized in field parasitology due to its practicality, and higher-resolution assays such as multiplex PCR could enhance detection performance in future work. Additionally, information on tick infestation, including species composition and infestation intensity, was not obtained since vector analysis was outside the scope of this investigation focused on molecular detection in goats. Future investigations with broader scale and integrated vector assessment are therefore recommended to better clarify transmission patterns and host–vector interactions.

## 5 Conclusion

This study provides exploratory evidence of a high prevalence of anaplasmosis and theileriosis in goats in Upper Egypt and reports the emerging zoonotic pathogens *Anaplasma capra* and *Theileria orientalis*. The combined microscopic and molecular approach highlights the value of integrated diagnostics in field epidemiology, contributing to the understanding of tick-borne diseases in goats in Egypt and emphasizing the need for continued surveillance and farmer awareness to support early detection, vigilant disease monitoring, and implementation of integrated control strategies against this pathogen. Future large scale investigations incorporating tick collection and molecular characterization of pathogens within vectors would further enhance understanding of host–vector relationships and overall disease transmission dynamics.

## Data Availability

The datasets presented in this study can be found in online repositories. The names of the repository/repositories and accession number(s) can be found in the article/supplementary material.

## Footnotes

1
www.totallab.com

2
https://blast.ncbi.nlm.nih.gov/Blast
